# Supplementary material for: Molecular chlamydia and gonorrhoea point of care tests implemented into routine practice: Systematic review and value proposition development
Source: PLoS One. 2021 Nov 8;16(11):e0259593. doi: 10.1371/journal.pone.0259593 (PMC8575247; doi:10.1371/journal.pone.0259593)
Supplement: S1 Table — (DOCX) [file pone.0259593.s001.docx]

|  | Terms |
| --- | --- |
| 1 | “Chlamydia” OR “Chlamydia trachomatis” OR “C. trachomatis” |
| 2 | “Gonorrh*” OR “Neisseria gonorrhoeae” OR “N. gonorrhoeae” |
| 3 | “point-of-care” OR “point of care” or “POCT” OR “POC” OR “rapid test*” OR “rapid diagn*” OR “rapid assay*” OR “near patient” OR “near-patient” OR “cepheid” OR “genexpert” OR “binx” OR “xpert” |
| 4 | Exp Point-of-care Testing/ |
| 5 | “Implement*” OR “evaluat*” OR “impact” OR “intervent*” OR “feasib*” OR “acceptab*” OR “effective*” OR “effic*” |
| 6 | 3 OR 4 |
| 7 | 1 and 2 and 5 and 6 |
| 8 | Limit 7 to (humans) |

**S1 Table:** **full list of search terms**
