# Supplementary material for: Molecular chlamydia and gonorrhoea point of care tests implemented into routine practice: Systematic review and value proposition development
Source: PLoS One. 2021 Nov 8;16(11):e0259593. doi: 10.1371/journal.pone.0259593 (PMC8575247; doi:10.1371/journal.pone.0259593)
Supplement: S3 Table — (DOCX) [file pone.0259593.s003.docx]

| **Article Information** | **Article Type** | **Study type** | **Score [meets criteria/total] (%)** | **Table reference** |
| --- | --- | --- | --- | --- |
| Adams EJ, Ehrlich A, Turner KME, Shah K, et al. Mapping patient pathways and estimating resource use for point of care versus standard testing and treatment of chlamydia and gonorrhoea in genitourinary medicine clinics in the UK. BMJ Open. 2014; 4(7). http://dx.doi.org/10.1136/bmjopen-2014-005322 | Article | Economic study | 7/10 (70) | 4 |
| Badman SG, Vallely LM, Toliman P, Kariwiga G, et al. A novel point-of-care testing strategy for sexually transmitted infections among pregnant women in high-burden settings: results of a feasibility study in Papua New Guinea. BMC Infect Dis. 2016; 16:250. https://dx.doi.org/10.1186/s12879-016-1573-4 | Article | Service evaluation | 5/5 (100) | 3 and 4 |
| Bell SFE, Coffey L, Debattista J, Badman SG, et al. Peer-delivered point-of-care testing for Chlamydia trachomatis and Neisseria gonorrhoeae within an urban community setting: A cross-sectional analysis. Sexual Health. 2020;17(4):359-67. http://dx.doi.org/10.1071/SH19233 | Article | Service evaluation | 5/5 (100) | 3 and 4 |
| Bristow CC, Mathelier P, Ocheretina O, Benoit D, et al. Chlamydia trachomatis, Neisseria gonorrhoeae, and Trichomonas vaginalis screening and treatment of pregnant women in Port-au-Prince, Haiti. Int J STD AIDS 2017;28(11):1130-4. http://dx.doi.org/10.1177/0956462416689755 | Article | Service evaluation | 5/5 (100) | 3 and 4 |
| Bourgeois-Nicolaos N, Jaureguy F, Pozzi-Gaudin S, Masson C, et al. Benefits of Rapid Molecular Diagnosis of Chlamydia Trachomatis and Neisseria Gonorrhoeae Infections in Women Attending Family Planning Clinics. Sex Transm Dis. 2015; 42(11):652-3. https://pubmed.ncbi.nlm.nih.gov/26462191/ | Article | Service evaluation | 4/5 (80) | 3 and 4 |
| Cohen S, Kohn R, Bacon O, De La Roca R, et al. Implementation of point of care gonorrhea and chlamydia testing in an std clinic prep program, san francisco, 2017-2018. Sex Transm Infect. 2019;95 (Suppl 1):A71-A2. http://dx.doi.org/10.1136/sextrans-2019-sti.185 | Abstract | Service evaluation | 5/5 (100) | 3 and 4 |
| Gaydos CA, Ako MC, Lewis M, Hsieh YH, et al. Use of a Rapid Diagnostic for Chlamydia trachomatis and Neisseria gonorrhoeae for Women in the Emergency Department Can Improve Clinical Management: Report of a Randomized Clinical Trial. Ann Emerg Med. 2019;74(1):36-44. http://dx.doi.org/10.1016/j.annemergmed.2018.09.012 http://dx.doi.org/10.1016/j.annemergmed.2018.09.012 | Article | Randomised Controlled Trial (RCT) | 9/10 (90) | 3 and 4 |
| Garrett NJ, Osman F, Maharaj B, Naicker N, et al. Beyond syndromic management: Opportunities for diagnosis-based treatment of sexually transmitted infections in low- and middle-income countries. PLoS One. 2018;13(4). http://dx.doi.org/10.1371/journal.pone.0196209 | Article | Service evaluation | 5/5 (100) | 3 and 4 |
| Gettinger J, Van Wagoner N, Daniels B, Boutwell A, et al. Patients Are Willing to Wait for Rapid Sexually Transmitted Infection Results in a University Student Health Clinic. Sex Transm Dis. 2020; 47(1):67-9. http://dx.doi.org/10.1097/OLQ.0000000000001083 | Article | Questionnaire study | 6/12 (50) | 4 |
| Guy RJ, Ward J, Causer LM, Natoli L, et al. Molecular point-of-care testing for chlamydia and gonorrhoea in Indigenous Australians attending remote primary health services (TTANGO): a cluster-randomised, controlled, crossover trial. Lancet Infect Dis. 2018;18(10):1117-26. http://dx.doi.org/10.1016/S1473-3099%2818%2930429-8 | Article | RCT | 10/10 (100) | 3 and 4 |
| Harding-Esch EM, Nori AV, Hegazi A, Pond MJ, et al. Impact of deploying multiple point-of-care tests with a sample first' approach on a sexual health clinical care pathway. A service evaluation. Sex Transm Infect. 2017;93(6):424-9. http://dx.doi.org/10.1136/sextrans-2016-052988 | Article | Service evaluation | 5/5 (100) | 3 and 4 |
| Hesse EA, Widdice LE, Patterson-Rose SA, St. Cyr S, et al. Feasibility and acceptability of point-of-care testing for sexually transmissible infections among men and women in mobile van settings. Sex Health. 2015;12(1):71-3. http://dx.doi.org/10.1071/SH14132 | Article | Service evaluation | 4/5 (80) | 3 and 4 |
| Keizur EM, Goldbeck C, Vavala G, Romero-Espinoza A, et al. Safety and Effectiveness of Same-Day Chlamydia trachomatis and Neisseria gonorrhoeae Screening and Treatment among Gay, Bisexual, Transgender, and Homeless Youth in Los Angeles, California, and New Orleans, Louisiana. Sex Transm Dis. 2020;47(1):19-23. http://dx.doi.org/10.1097/OLQ.0000000000001088 | Article | Service evaluation | 5/5 (100) | 3 and 4 |
| Kerry-Barnard S, Huntington S, Fleming C, Reid F, et al. Near patient chlamydia and gonorrhoea screening and treatment in further education/technical colleges: a cost analysis of the 'Test n Treat' feasibility trial. BMC Health Serv Res. 2020; 20(1):316. http://dx.doi.org/10.1186/s12913-020-5062-5 | Article | Economic study | 6/10 (60) | 4 |
| Martin K, Olaru ID, Buwu N, Bandason T, et al. Uptake of and factors associated with testing for sexually transmitted infections in community-based settings among youth in Zimbabwe: a mixed-methods study. Lancet Child Adolesc Health 2021; 5: 122–32 https://doi.org/10.1016/S2352-4642(20)30335-7 | Article | Service evaluation | 5/5 (100) | 3 and 4 |
| Mandlik E, Plaha K, Jones R, Rayment M. Cutting the time to treatment of *chlamydia trachomatis* (CT) and *neisseria gonorrhoeae* (NG) with near-patient molecular diagnostics: The utility of the Cepheid Gene Xpert system. Sex Transm Infect. 2017; 93 (Suppl 1):A50. http://dx.doi.org/10.1136/sextrans-2017-053232.145 | Abstract | Before and after study | 2/8 (25) | 3 and 4 |
| Mvumbi G, Hoff NA, Musene K, Gadoth A, et al. Implication of using the GeneXpert for the detection of STIs in pregnant women in Kisantu Health Zone, DRC. Trop Med Int Health. 2017; 22 (Suppl 1):170. https://onlinelibrary.wiley.com/doi/epdf/10.1111/tmi.12979 | Abstract | Service evaluation | 2/5 (40) | 3 and 4 |
| May, L, Ware, CE, Jeanne JA, Zocchi M, et al. A Randomized Controlled Trial Comparing the Treatment of Patients Tested for Chlamydia and Gonorrhea After a Rapid Polymerase Chain Reaction Test Versus Standard of Care Testing. Sex Transm Dis. 2016; (5)43. https://journals.lww.com/stdjournal/Fulltext/2016/05000/A_Randomized_Controlled_Trial_Comparing_the.4.aspx | Article | RCT | 10/10 (100) | 3 and 4 |
| Natoli L, Guy RJ, Shephard M, Causer L, et al. "I do feel like a scientist at times": A qualitative study of the acceptability of molecular point-of-care testing for chlamydia and gonorrhoea to primary care professionals in a remote high STI burden setting. PLoS One. 2015; 10(12). http://dx.doi.org/10.1371/journal.pone.0145993 | Article | Qualitative study | 8/9 (88.8) | 4 |
| Rivard KR, Dumkow LE, Draper HM, Brandt KL, et al. Impact of rapid diagnostic testing for chlamydia and gonorrhea on appropriate antimicrobial utilization in the emergency department. Diagn Microbiol Infect Dis. 2017;87(2):175-9. http://dx.doi.org/10.1016/j.diagmicrobio.2016.10.019 | Article | Before and after study | 7/8 (87.5) | 3 and 4 |
| Skaletz-Rorowski A, Potthoff A, Nambiar S, Wach J, et al. Sexual behaviour, STI knowledge and Chlamydia trachomatis (CT) and Neisseria gonorrhoeae (NG) prevalence in an asymptomatic cohort in Ruhr-area, Germany: PreYoungGo study. J Eur Acad Dermatol Venereol. 2020; 35: 241-246. http://dx.doi.org/10.1111/jdv.16913 | Article | Service evaluation | 3/5 (60) | 3 and 4 |
| Turner KME, Ound J, Horner P, Macleod J, et al. An early evaluation of clinical and economic costs and benefits of implementing point of care naat tests for chlamydia trachomatis and neisseria gonorrhoea in genitourinary medicine clinics in england. Sex Transm Infect. 2014; 90(2):104-11. http://dx.doi.org/10.1136/sextrans-2013-051147 | Article | Economic study | 9/11 (81.8) | 4 |
| Whitlock G, Byrne R, Cooper F, McOwan A. A novel model of care incorporating self-directed care and rapid results management successfully reaches high-risk men who have sex with men. Int J STD AIDS. 2015; 26(11):88. http://dx.doi.org/10.1177/0956462415601768 | Abstract | Service evaluation | 1/5 (20) | 3 and 4 |
| Whitlock GG, Gibbons DC, Longford N, Harvey MJ, et al. Rapid testing and treatment for sexually transmitted infections improve patient care and yield public health benefits. Int J STD AIDS. 2018; 29(5):474-82. http://dx.doi.org/10.1177/0956462417736431 | Article | Service evaluation | 5/5 (100) | 3 and 4 |
| Widdice LE, Hsieh YH, Silver B, Barnes M, et al. Performance of the Atlas Genetics Rapid Test for Chlamydia trachomatis and Women's Attitudes Toward Point-Of-Care Testing. Sex Transm Dis. 2018; 45(11):723-7. https://dx.doi.org/10.1097/OLQ.0000000000000865 | Article | Questionnaire study | 9/12 (75) | 4 |
| Wingrove I, McOwan A, Nwokolo N, Whitlock G. Diagnostics within the clinic to test for gonorrhoea and chlamydia reduces the time to treatment: A service evaluation. Sex Transm Infect. 2014; 90(6):474. http://dx.doi.org/10.1136/sextrans-2014-051580 | Article | Service evaluation | 3/5 (60) | 3 and 4 |
| Wynn A, Ramogola-Masire D, Gaolebale P, Moshashane N, et al. Acceptability and Feasibility of Sexually Transmitted Infection Testing and Treatment among Pregnant Women in Gaborone, Botswana, 2015. Biomed Res Int. 2016:6. http://dx.doi.org/10.1155/2016/1251238 | Article | Service evaluation | 4/4 (100) | 3 and 4 |
| Wynn A, Moucheraud C, Morroni C, Ramogola-Masire D, et al. Antenatal testing for curable STIs compared to syndromic management in Botswana: A cost-effectiveness study. Sex Transm Infect. 2019; 95 (Suppl 1):A90. http://dx.doi.org/10.1136/sextrans-2019-sti.231 | Abstract | Economic study | 5/11 (45.4) | 4 |
