# Supplementary material for: Molecular chlamydia and gonorrhoea point of care tests implemented into routine practice: Systematic review and value proposition development
Source: PLoS One. 2021 Nov 8;16(11):e0259593. doi: 10.1371/journal.pone.0259593 (PMC8575247; doi:10.1371/journal.pone.0259593)
Supplement: S4 Table — (DOCX) [file pone.0259593.s004.docx]

| **Articles** | **Criteria for assessment** | | | | | |
| --- | --- | --- | --- | --- | --- | --- |
|  | Were the criteria for inclusion in the sample clearly defined? | Were the study subjects and the setting described in detail? | Were objective, standard criteria used for measurement of the condition? | Were the outcomes measured in a valid and reliable way? | Was appropriate statistical analysis used? | Score |
| Badman et al 2016 | Y | Y | Y | Y | Y | 5/5 |
| Bell et al 2020 | Y | Y | Y | Y | Y | 5/5 |
| Bourgeois-Nicolaos et al 2015 | Y | Y | Y | Y | N | 4/5 |
| Bristow et al. 2017. | Y | Y | Y | Y | Y | 5/5 |
| Cohen et al 2019 | Y | Y | Y | Y | Y | 5/5 |
| Garrett et al. 2018. | Y | Y | Y | Y | Y | 5/5 |
| Harding-Esch et al. 2017. | Y | Y | Y | Y | Y | 5/5 |
| Hesse et al. 2015. | Y | U | Y | Y | Y | 4/5 |
| Keizur et al. 2020. | Y | Y | Y | Y | Y | 5/5 |
| Martin et al. 2021 | Y | Y | Y | Y | Y | 5/5 |
| Mvumbi et al 2019 | U | N | Y | Y | N | 2/5 |
| Skaletz-Rorowski et al 2020 | N | N | Y | Y | Y | 3/5 |
| Whitlock et al. 2018. | Y | Y | Y | Y | Y | 5/5 |
| Whitlock et al. 2015 | N | N | Y | U | U | 1/5 |
| Wingrove et al. 2014. | U | U | Y | Y | Y | 3/5 |
| Wynn et al 2016 | Y | Y | Y | Y | N/A | 4/4 |
