# Supplementary material for: Molecular chlamydia and gonorrhoea point of care tests implemented into routine practice: Systematic review and value proposition development
Source: PLoS One. 2021 Nov 8;16(11):e0259593. doi: 10.1371/journal.pone.0259593 (PMC8575247; doi:10.1371/journal.pone.0259593)
Supplement: S5 Table — (DOCX) [file pone.0259593.s005.docx]

|  | Mandlik et al 2017 | Rivard et al 2017 |
| --- | --- | --- |
| Was the study question or objective clearly stated? | N | Y |
| Were eligibility/selection criteria for the study population prespecified and clearly described? | N | Y |
| Were the participants in the study representative of those who would be eligible for the test/service/intervention in the general or clinical population of interest? | Cannot determine | Y |
| Were all eligible participants that met the prespecified entry criteria enrolled? | Cannot determine | Cannot determine |
| Was the sample size sufficiently large to provide confidence in the findings? | Y | Y |
| Was the test/service/intervention clearly described and delivered consistently across the study population? | Cannot determine | Y |
| Were the outcome measures prespecified, clearly defined, valid, reliable, and assessed consistently across all study participants? | Y | Y |
| Were the people assessing the outcomes blinded to the participants' exposures/interventions? | N/A | N/A |
| Was the loss to follow-up after baseline 20% or less? Were those lost to follow-up accounted for in the analysis? | N/A | N/A |
| Did the statistical methods examine changes in outcome measures from before to after the intervention? Were statistical tests done that provided p values for the pre-to-post changes? | N | Y |
| Were outcome measures of interest taken multiple times before the intervention and multiple times after the intervention (i.e., did they use an interrupted time-series design)? | N/A | N/A |
| If the intervention was conducted at a group level (e.g., a whole hospital, a community, etc.) did the statistical analysis take into account the use of individual-level data to determine effects at the group level? | N/A | N/A |
| Score | 2/8 | 7/8 |
