# Supplementary material for: Molecular chlamydia and gonorrhoea point of care tests implemented into routine practice: Systematic review and value proposition development
Source: PLoS One. 2021 Nov 8;16(11):e0259593. doi: 10.1371/journal.pone.0259593 (PMC8575247; doi:10.1371/journal.pone.0259593)
Supplement: S6 Table — (DOCX) [file pone.0259593.s006.docx]

|  | Gaydos at al 2019 | Guy et al 2018 | May et al 2016 |
| --- | --- | --- | --- |
| Was true randomization used for assignment of participants to treatment groups? | Y | Y | Y |
| Was allocation to treatment groups concealed? | Y | N | Y |
| Were treatment groups similar at the baseline? | Y | Y | Y |
| Were participants blind to treatment assignment? | N/A | N/A | N/A |
| Were those delivering treatment blind to treatment assignment? | N/A | N/A | N/A |
| Were outcomes assessors blind to treatment assignment? | N/A | N/A | N/A |
| Were treatment groups treated identically other than the intervention of interest? | Y | Y | Y |
| Was follow up complete and if not, were differences between groups in terms of their follow up adequately described and analyzed? | Y | Y | Y |
| Were participants analyzed in the groups to which they were randomized? | Y | Y | Y |
| Were outcomes measured in the same way for treatment groups? | Y | Y | Y |
| Were outcomes measured in a reliable way? | Y | Y | Y |
| Was appropriate statistical analysis used? | U | Y | Y |
| Was the trial design appropriate, and any deviations from the standard RCT design (individual randomization, parallel groups) accounted for in the conduct and analysis of the trial? | Y | Y | Y |
| Score | 9/10 | 9/10 | 10/10 |
