# Supplementary material for: Molecular chlamydia and gonorrhoea point of care tests implemented into routine practice: Systematic review and value proposition development
Source: PLoS One. 2021 Nov 8;16(11):e0259593. doi: 10.1371/journal.pone.0259593 (PMC8575247; doi:10.1371/journal.pone.0259593)
Supplement: S7 Table — (DOCX) [file pone.0259593.s007.docx]

| **Criteria for assessment** | **Article** |
| --- | --- |
|  | Natoli et al 2015 |
| Is there congruity between the stated philosophical perspective and the research methodology? | N/A |
| Is there congruity between the research methodology and the research question or objectives? | Y |
| Is there congruity between the research methodology and the methods used to collect data? | Y |
| Is there congruity between the research methodology and the representation and analysis of data? | Y |
| Is there congruity between the research methodology and the interpretation of results? | Y |
| Is there a statement locating the researcher culturally or theoretically? | N |
| Is the influence of the researcher on the research, and vice- versa, addressed? | Y |
| Are participants, and their voices, adequately represented? | Y |
| Is the research ethical according to current criteria or, for recent studies, and is there evidence of ethical approval by an appropriate body? | Y |
| Do the conclusions drawn in the research report flow from the analysis, or interpretation, of the data? | Y |
