# Supplementary material for: Molecular chlamydia and gonorrhoea point of care tests implemented into routine practice: Systematic review and value proposition development
Source: PLoS One. 2021 Nov 8;16(11):e0259593. doi: 10.1371/journal.pone.0259593 (PMC8575247; doi:10.1371/journal.pone.0259593)
Supplement: S8 Table — (DOCX) [file pone.0259593.s008.docx]

| **Criteria for assessment** | **Articles** | |
| --- | --- | --- |
|  | Gettinger et al 2020 | Widdice et al 2018 |
| Did the study address a clearly focused question / issue? | Y | Y |
| Is the research method (study design)appropriatefor answering the research question? | Y | Y |
| Is the method of selection of the subjects (employees, teams, divisions, organizations) clearly described? | N | Y |
| Has the way the sample was obtained avoid (selection)bias?* | Can't tell | Y |
| Was the sample of subjects representative with regard to the population to which the findings will be referred? | Can't tell | Can't tell |
| Was the sample size based on pre-study considerations of statistical power? | Can't tell | Can't tell |
| Was a satisfactory response rate achieved? | Y | Y |
| Are the measurements (questionnaires) likely to be valid and reliable? | Y | Y |
| Was the statistical significance assessed? | Y | Y |
| Are confidence intervals given for the main results? | N | Y |
| Have all confounding factors been accounted for?** | N | N |
| Can the results be applied to your organization? | Y | Y |

*Original question: Could the way the sample was obtained introduce (selection)bias?

**Original question: Could there be confounding factors that haven’t been accounted for?
