# Supplementary material for: Molecular chlamydia and gonorrhoea point of care tests implemented into routine practice: Systematic review and value proposition development
Source: PLoS One. 2021 Nov 8;16(11):e0259593. doi: 10.1371/journal.pone.0259593 (PMC8575247; doi:10.1371/journal.pone.0259593)
Supplement: S9 Table — (DOCX) [file pone.0259593.s009.docx]

| **Criteria for assessment** | **Articles** | | | |
| --- | --- | --- | --- | --- |
|  | Adams et al 2014 | Turner et al 2013 | Wynn et al 2019 | Kerry-Barnard et al 2020 |
| Is there a well-defined question? | Y | Y | Y | Y |
| Is there comprehensive description of alternatives? | Y | Y | Y | Y |
| Are all important and relevant costs and outcomes for each alternative identified? | Y | Y | unclear | Y |
| Has clinical effectiveness been established? | N/A | Y | Y | N/A |
| Are costs and outcomes measured accurately? | Y | Y | unclear | Y |
| Are costs and outcomes valued credibly? | Y | Y | unclear | Y |
| Are costs and outcomes adjusted for differential timing? | N | N | N | N |
| Is there an incremental analysis of costs and consequences? | Y | Y | N | N/A |
| Were sensitivity analyses conducted to investigate uncertainty in estimates of cost or consequences? | N | Y | Y | N |
| Do study results include all issues of concern to users? | N | N | N | N |
| Are the results generalizable to the setting of interest in the review? | Y | Y | Y | Y |
